# HLA与肺癌免疫

**DOI:** 10.3779/j.issn.1009-3419.2010.02.14

**Published:** 2010-02-20

**Authors:** 慧娟 王, 秦秦 王

**Affiliations:** 650032 昆明，昆明医学院第一附属医院临床实验研究中心 Clinical Medicine Research Center of First Hospital of Kunming Medical College, Kunming 650032, China

人类白细胞抗原(human leukocyte antigen, HLA)在机体细胞免疫过程中参与抗原分子的提呈, 在机体抗肿瘤免疫机制中占有重要地位, 目前有大量关于HLA与肺癌发生发展之间关系的研究, 并对此提出了一些可能的治疗方法, 为肿瘤的免疫治疗提供了新的思路。

## 1 HLA

引起急性移植排斥反应的同种异型抗原称为主要组织相容性抗原(major histocompatibility antigen), 编码这组抗原的基因称为主要组织相容性复合体(major histocompatibility complex, MHC)。人类主要组织相容性抗原是在白细胞中发现的, 所以又称为HLA。

### 1.1 HLA复合体的结构

编码HLA的基因群又称为HLA复合体, 定位于人类第6号染色体短臂q21.31和q21.32区之间, 分为HLA-Ⅰ类、HLA-Ⅱ类和HLA-Ⅲ类3个区, 跨度约为4 000 kb, 约包含450个基因。其中经典的HLA基因一般专指与组织相容性有关的、具有向T细胞提呈蛋白质抗原功能的基因。

HLA-Ⅰ类区中约包含3个经典的HLA-Ⅰ类基因, 即HLA-A、HLA-B、HLA-C。HLA-Ⅱ类区包括HLA-DP、HLA-DQ、HLA-DR三个经典HLA-Ⅱ类基因及一些与抗原处理和提呈有关的基因, 包括TAP1、TAP2、LMP2等。HLA-Ⅲ类区包含75个基因, 大多数功能不明, 其中一小部分是与HLA无关的、编码分泌型蛋白的基因, 其产物是免疫系统的重要成分。

### 1.2 HLA分子的结构和功能

HLA-Ⅰ类和HLA-Ⅱ类分子是细胞表面跨膜糖蛋白, 又称为HLA-Ⅰ类和HLA-Ⅱ类抗原。HLA-Ⅰ类分子广泛分布于体内有核细胞膜表面, 以淋巴细胞表面密度最大。与HLA-Ⅰ类分子相比, HLA-Ⅱ类分子的表达更为局限, 正常情况下主要表达在专职抗原提呈细胞表面, 主要包括B细胞、单核巨噬细胞、树突状细胞。

HLA-Ⅰ类分子是由一条重链和一条轻链通过非共价结合形成的异二聚体。重链又称α链, 由经典的HLA-A、HLA-B、HLA-C基因编码; 轻链为β_2_微球蛋白(β_2_ m), 其编码基因位于第15号染色体。HLA-Ⅱ类分子是由α链和β链组成的异二聚体。α链和β链的编码基因均位于HLA-Ⅱ类区的D区内。

HLA-Ⅰ类分子的主要功能是作为杀伤性CD8^+^T细胞的识别标志之一, 参与内源性抗原的提呈。HLA-Ⅱ类分子作为CD4^+^T细胞的识别标志之一, 主要参与外源性抗原的递呈。另外, 一些HLA-Ⅰ类分子, 包括非经典的Ⅰ类分子HLA-E和HLA-G, 还可以与NK细胞表面的受体结合, 影响NK细胞的功能。HLA分子由于直接影响机体的细胞免疫, 因而成为免疫应答过程中的关键分子^[1]^。

## 2 机体抗肿瘤免疫效应机制中HLA的作用

机体具有强大的免疫功能, 通过免疫监视可以发现并且杀伤肿瘤细胞。机体抗肿瘤的免疫效应是多途径的, 一般认为细胞免疫比体液免疫在抗肿瘤效应中发挥着更重要的作用。

抗肿瘤的效应细胞以HLA-Ⅰ类抗原限制的CD8^+^细胞毒性T细胞(cytotoxic T lymphocyte, CTL)和HLA-Ⅱ类抗原限制的CD4^+^辅助性T细胞(helper T lymphocyte, Th)为主。CD8^+^CTL活化增殖后, 直接杀伤肿瘤细胞, 或者通过分泌细胞因子发挥抗肿瘤作用, 是最主要的抗肿瘤细胞。CD4^+^T细胞活化后可产生淋巴因子, 增强CTL和自然杀伤细胞(natural killer cell, NK cell)的功能并可激活巨噬细胞或其它抗原提呈细胞(antigen-presenting cell, APC)参与抗肿瘤作用。NK细胞也参与抗肿瘤作用且不受MHC限制。未活化的NK细胞仅可杀伤某些肿瘤细胞, 活化后杀瘤效率大幅提高。干扰素(interferon, IFN)、白介素2(interleukin, IL-2)等细胞因子可以强化其功能。NK细胞主要通过抗体依赖性细胞介导的细胞毒作用杀伤肿瘤细胞, 而其杀伤肿瘤细胞的活性受活化性受体和抑制性受体调节。HLA-B、HLA-C、HLA-E和HLA-G等编码的基因产物可以识别NK细胞抑制性受体, 抑制NK细胞的杀伤作用。γδT细胞大多数为CD4^–^CD8^–^T细胞, 具有非MHC限制性的杀伤肿瘤细胞作用。另外, 巨噬细胞、活化的自然杀伤T细胞、淋巴细胞激活的杀伤细胞和肿瘤浸润的淋巴细胞也具有抗肿瘤作用。

因此, HLA-Ⅰ类分子的正常表达保证了对CTL的抗原提呈和NK细胞的正常杀伤活性, 使机体处于正常免疫状态之下。若细胞表面的HLA分子异常表达, 就可能使这种异常细胞逃避免疫监视而影响机体的健康。

## 3 肺癌患者HLA基因表达的相关研究

### 3.1 肺肿瘤细胞HLA-Ⅰ类基因的异常表达

近年来大量的临床研究已经证实, 经典HLA-Ⅰ类抗原的表达在多种恶性肿瘤中均明显降低。由于CTL的MHC限制性, 肿瘤细胞表面的HLA-Ⅰ类分子表达下调甚至缺失将导致T细胞不能被刺激活化, 不能产生有效的细胞免疫应答。而肿瘤细胞异常高表达HLA-E、HLA-G, 则会抑制NK细胞的杀伤活性。

目前在肺癌的相关研究中, 研究对象多集中于非小细胞肺癌(non-small cell lung cancer, NSCLC), 而关于经典的HLA-Ⅰ类抗原的表达情况与预后的关系, 尚未取得完全一致的结论。

Baba等^[2]^研究了26种肺癌细胞株中HLA-Ⅰ类抗原的情况, 他们发现10种有HLA-Ⅰ类基因单倍型缺失, 3种有HLA-Ⅰ类抗原表达缺失。Kikuchi等^[3]^在161例NSCLC组织中发现, 有68.9%的癌组织HLA-Ⅰ类抗原低表达或者表达缺失, 而且这部分癌组织中CD8^+^T细胞量明显少于HLA-Ⅰ类抗原强阳性的癌组织中的CD8^+^T细胞量; HLA-Ⅰ类抗原的低表达还可以作为病理1期的患者预后不良的指标。Suzuki等^[4]^在105例NSCLC组织中发现58.3%有经典的HLA-Ⅰ类分子缺失, 并且发现HLA-Ⅰ类分子与肿瘤的大小、瘤体的发展和病理分期相关。Ramnath等^[5]^发现HLA-Ⅰ类分子缺失在NSCLC中十分普遍(93.6%), 但在体内HLA-Ⅰ类分子的表达情况与NSCLC的生存率无统计学关联, 认为NSCLC中NK细胞是主要的抗肿瘤细胞。另外, 王威等^[6]^研究发现HLA-A、HLA-B、HLA-C抗原在鳞癌中表达下降率明显高于腺癌(*P* < 0.05)。

与上述观点不同的是, Nagata等^[7]^在研究了695例NSCLC组织HLA-Ⅰ类分子等位基因后得出结论, 病理1期HLA-A2阴性患者的预后要好于HLA-A2阳性的患者; 而在病理2、3期的患者中, HLA-A24阳性可以作为不良预后的指标。

在非经典的HLA-Ⅰ类分子的研究中, HLA-G在多种肿瘤中的异常高表达也引起了研究者们的注意, 认为HLA-G抑制了NK细胞杀伤肿瘤的能力。Yie等^[8]^在NSCLC组织中发现75%的癌组织中有HL A-G的异常表达, HLA-G高表达的患者预后较差; HLA-G的异常表达与淋巴结转移、临床分期和患者本身的免疫应答效力相关; 并且HLA-G高表达可以作为NSCLC的特征之一。

经典的HLA-Ⅰ类抗原在肿瘤中表达下调的机制尚不十分清楚。染色体缺失、体细胞重组、基因突变及转录调节和蛋白运输等环节障碍都有可能参与恶性肿瘤HLA基因的异常表达。Paschen等^[9]^对黑色素瘤细胞研究后认为, HLA-Ⅰ类基因缺失是15号染色体失稳定和β_2_微球蛋白基因突变同时发生引起的。Corrias等^[10]^则认为HLA-Ⅰ类分子重链、β_2_微球蛋白、TAP-1和TAP-2表达缺失等多种机制是神经胶质瘤细胞HLA-Ⅰ类抗原表达低下的原因。Maleno等^[11]^研究表明喉癌细胞HLA-Ⅰ类基因缺失有多种类型, 如HLA-Ⅰ类基因全部缺失、HLA-Ⅰ类基因单倍型选择性缺失、HLA-A或HLA-B基因位点的选择性降低、单个HLA-Ⅰ类等位基因的选择性缺失或降低等。HLA基因单倍型缺失是引起肿瘤HLA表型改变的最常见的原因。对肺肿瘤研究后So等^[12]^认为HLA-Ⅰ类基因表达下调或者缺失的肿瘤类型是T细胞免疫选择的结果, 而且这种HLA-Ⅰ类基因的异常表达是由HLA-Ⅰ类基因单倍型缺失引起的。邓波等^[13]^研究后认为, HLA-Ⅰ类基因单倍型选择性丢失可能是肿瘤细胞在传代过程中姐妹染色体不分离或染色体重组错误如染色体缺失等造成的。而庄东刚等^[14]^实验结果提示HLA-Ⅰ类分子的基因结构出现改变的机率较小, 推测HLA-Ⅰ类分子在肿瘤中表达的缺失可能是相关基因在从转录到翻译的过程中的某一环节或者是某些环节受到致癌因素的影响, 进而导致HLA-Ⅰ类分子表达下调或表达缺失。

从上述的研究结果来看, HLA-Ⅰ类基因在肺癌组织中表达的情况在各个研究中的结果并不完全一致, 异常表达的机制仍需要进一步研究。

### 3.2 肺癌患者HLA-Ⅱ类基因的异常表达

近年在人的多种恶性组织的研究中均发现有HLA-Ⅱ类基因的异常表达。大量的研究显示肿瘤细胞HLA-Ⅱ类基因的表达可以使肿瘤细胞具有抗原递呈、活化Th细胞的作用, 引起抗肿瘤效应。Matsushita等^[15]^在预后良好的结肠癌患者的癌组织中发现有HLA-DR的高表达, 并且可以作为独立的预后良好的指标。但有研究^[16]^认为若肿瘤细胞不表达其它共刺激因子, 则可以导致免疫耐受, 认为其作用取决于表达的量。因此目前肿瘤细胞HLA-Ⅱ类基因表达对机体免疫的影响还存在争议。肿瘤细胞异常表达HLA-DR的机制目前尚不十分清楚。van der Stoep等^[17]^发现HLA-Ⅱ类基因反式作用因子(class Ⅱ transactivator, C Ⅱ TA)启动子Ⅳ可以与MHC Ⅱ类基因相互作用而刺激HLA-Ⅱ类分子的表达; IFN_-γ_可以激活C Ⅱ TA启动子Ⅳ, 而许多肿瘤细胞IFN_-γ_诱导HLA-Ⅱ类分子表达的现象消失了。在对神经胶质瘤细胞研究后他们发现, C Ⅱ TA启动子Ⅳ发生了甲基化, 认为肿瘤细胞在发展早期通过甲基化C Ⅱ TA启动子Ⅳ抑制HLA-Ⅱ类分子的表达, 而分化后的肿瘤细胞则没有这种甲基化现象。

在肺癌的相关研究中, Deng等^[18]^在两种肺癌细胞株中发现保存有完整的HLA-Ⅱ类基因。Kataki等^[19]^对48例NSCLC组织进行了研究, 发现仅有8%有HLA-Ⅱ类分子的表达。而Pouniotis等^[20]^在对4种肺癌类型(腺癌、鳞癌、大细胞未分化癌、小细胞肺癌)研究后发现, 仅有小细胞肺癌组织有HLA-Ⅱ类分子的低表达。马晓春等^[21]^在研究中发现, 肺癌细胞的HLA-DR抗原阳性率为43%, 腺癌的阳性率明显高于鳞癌和小细胞癌。他们还观察到HLADR抗原染色较强的癌细胞周围有较多的淋巴细胞浸润。他们推测是淋巴细胞浸润释放出的如γ-干扰素之类的淋巴因子引起了肺癌细胞的HLA-DR抗原表达。然而, 部分病例癌组织中虽有较多的淋巴细胞浸润, 但却不表达HLA-DR抗原。肺癌细胞表达HLA-DR抗原与其病理、预后关系等方面仍需要进一步研究。

### 3.3 肺肿瘤患者外周血T淋巴细胞HLA-DR的表达降低

陈清勇等^[22]^应用流式细胞双荧光染色技术发现肺癌组外周T血淋巴细CD3^+^/HLA-DR^+^的表达水平明显低于正常对照组。杨凌等^[23]^也得到相同的结论, 并发现正常对照组和良性病变组之间无明显差异; 随着肿瘤进展和年龄的增高, 肺癌患者外周血T淋巴细胞中CD3^+^/HLA-DR^+^的表达水平逐渐降低; 中、高分化程度的肺癌患者的CD3^+^/HLA-DR^+^表达明显高于低分化程度的肺癌; 肺癌伴淋巴结转移者的外周血CD3^+^/HLA-DR^+^的表达水平明显低于无淋巴结转移者。他们认为这些变化可能是肺癌细胞产生一些免疫抑制因子, 通过抑制外周血中T淋巴细胞的活化功能, 如CD25^+^/HLA-DR^+^的表达水平, 来进一步抑制宿主的免疫应答, 从而使癌细胞逃避机体免疫监视。

## 4 针对肺癌患者HLA分子表达异常而提出的治疗方法

目前肿瘤的免疫治疗方法很多, 本文主要就针对HLA的治疗方法作一综述。研究者根据上述现象认为, 肿瘤免疫治疗的关键在于增强肿瘤细胞中HLA-Ⅰ类抗原的表达, 增强免疫系统识别肿瘤细胞的能力。通过免疫治疗增强肿瘤细胞表面的HLA-Ⅰ类抗原表达, 可能会取得较好的疗效。

### 4.1 基因治疗

20世纪90年代, 研究者已经发现, 将某些基因转染入肿瘤细胞后可以导致其HLA-Ⅰ类抗原表达升高, 致瘤性下降。

Torabi-Pour等^[24]^将β_2_微球蛋白基因利用脂质体转染技术导入膀胱癌细胞中, 发现HLA-Ⅰ类抗原表达明显上调, 并且IFN可以诱导这些细胞HLA-Ⅰ、HLA-Ⅱ类抗原的表达。Salamone等^[25]^利用载体将同种异体HLA-B7基因和β_2_微球蛋白基因共转染入HLA-B7缺失的头颈部肿瘤细胞, 与转染自体HLA-B7基因相比, 可以更有效地诱导HLA Ⅰ类抗原的表达, 抑制肿瘤生长。Lou等^[26]^将重组有TAP1基因的腺病毒转入HLA抗原表达通路缺陷的鼠肺癌细胞系CMT 64后发现TAP1的表达可以提高细胞表面HLA抗原的表达; 该研究结果也证实, 参与HLA分子组装和运输表达过程的分子异常也可以导致HLA抗原的低表达。

### 4.2 细胞因子干扰素(IFN)及组蛋白脱乙酰基酶(histone deacetylase, HDAC)抑制子

Merritt等^[27]^发现无论在体外还是体内, IFN_-γ_都可以诱导肿瘤细胞HLA-Ⅰ类分子的表达。Read等^[28]^证实, 在体外IFN_-γ_可以诱导正常脑细胞和脑肿瘤细胞的表达, 其中HLA-Ⅰ类抗原明显增加, 而HLA-Ⅱ类抗原的表达也可以被诱导; 将*IFN_-γ_*基因利用逆转录病毒载体转入神经胶质瘤细胞后HLA-Ⅰ类和HLAⅡ类抗原的表达均增加。徐亦益等^[29]^将克隆的人源*IFN*基因转基因人肺腺癌细胞A549, 发现该转基因细胞能稳定且持久地向胞外分泌IFN蛋白, 并且该转基因瘤苗的HLA-Ⅰ、HLA-Ⅱ类分子的表达得到上调。以上研究均提示细胞因子IFN可以提高肿瘤细胞的免疫原性和提呈抗原的能力。

目前的研究^[16]^显示正常组织中的IFN_-γ_可以诱导HLA-Ⅱ类分子的表达, 而许多人类肿瘤细胞的HLA-Ⅱ类分子并不能被IFN_-γ_诱导产生。抑癌基因Rb蛋白的缺失与这种现象密切相关。HDAC抑制子MS-275可以重建_γ-_IFN对缺失Rb蛋白的NSCLC细胞的可诱导性, 提高HLA-Ⅱ类分子特别是HLA-DR的表达率。目前MS-275正处于NSCLC治疗的1、2期临床试验中^[30]^。

### 4.3 肿瘤细胞疫苗

Indrová等^[31]^将*IL-12*基因用腺病毒转导入肿瘤细胞制成肿瘤细胞疫苗, 再打入已成瘤的小鼠体内, 发现可使HLA-Ⅰ类分子表达增加。

### 4.4 其它

李兆忠等^[32]^采用反义核酸技术证实c-myc反义寡核苷酸可以上调人高转移肺癌细胞表面HLA-A、HLA-B、HLA-C、ICAM-1分子的表达水平。

综上所述, 目前研究均在肺癌患者的肺肿瘤细胞表面发现有HLA分子的异常表达, 并且这种异常与肺癌的类型、进展、预后等有关, 但是研究结果并不完全一致。在肺癌患者外周血中T淋巴细胞的表面也发现有HLA-DR的表达降低。目前可以肯定的是这些异常表达的HLA分子与肺癌细胞逃逸免疫监视有着密切的联系, 具体机制仍需要进一步的研究。基于以上研究结果, 研究者们还提出了增强肺肿瘤细胞HLA-Ⅰ类分子表达的免疫治疗方法, 虽然尚不成熟, 但为肿瘤的免疫治疗提供了新的方向。
